# A83 ADAPTATION AND VALIDATION OF QUESTIONNAIRES TO MEASURE SATISFACTION WITH TELEPHONE CARE AMONG IBD PATIENTS AND GASTROENTEROLOGY CARE PROVIDERS

**DOI:** 10.1093/jcag/gwac036.083

**Published:** 2023-03-07

**Authors:** J N Foncham, N Rohatinsky, S Fowler, J N Peña-Sánchez

**Affiliations:** 1 Community Health and Epidemiology; 2 College of Nursing; 3 College of Medicine, University osf Saskatchewan, Saskatoon, Canada

## Abstract

**Background:**

People living with inflammatory bowel disease (IBD) require regular medical follow-up, which could be challenging for individuals living in rural areas and those who have limited access to specialized care. Telephone care (TC) could improve health care by increasing access to specialized care. The Covid-19 pandemic resulted in increased use of virtual care, which was predominantly TC in Saskatchewan (SK) , Canada. There are no validated questionnaires to measure satisfaction with TC among IBD patients and gastrointestinal care providers (GCPs).

**Purpose:**

This study aimed to adapt and validate a questionnaire to evaluate the satisfaction of IBD individuals and GCPs with TC in SK, Canada.

**Method:**

The Telehealth Usability Questionnaire was adapted to the IBD TC context by a committee of experts, comprised of three IBD GCPs, two IBD-patient partners, and two health care researchers. Two questionnaires were generated -: the Telephone Care Satisfaction Questionnaire (TCSQ) for patients (IBD-TCSQ-patient) and GCPs (IBD-TCSQ-provider). The committee evaluated the content validity of the adapted questionnaires. A pilot assessed the readability and usability of the questionnaire items. Subsequently, individuals living with IBD in SK and GCPs completed an online survey with the TCSQ-patient and IBD-TCSQ-provider questionnaires in the winter of 2022. Data were analysed using descriptive and correlational techniques. Psychometric analyses were conducted to examine the reliability and validity of the TCSQ-patient, but not for the TCSQ-provider due to small sample size.

**Result(s):**

The IBD-TCSQ-patient and IBD-TCSQ-provider questionnaires were developed, each with 16 individual items and one question on global TC satisfaction. The pilot demonstrated good readability and usability of the questionnaires. Then, 87 IBD individuals completed the IBD-TCSQ-patient and six GCPs the IBD-TCSQ-provider. The standardized level of TC satisfaction for the 16-item IBD-TCSQ-patient was 5.70 (SD=0.94) on a scale from 1.00-7.00. All items of the IBD-TCSQ-patient were significantly correlated (p<0.001). A strong correlation was observed between the 16-item standardized TC satisfaction and its overall item r=0.85 (p<0.001). The IBD-TCSQ-patient had optimal internal reliability (α=0.96). Two factors were identified in the exploratory factor analysis. Factor 1 focused on TC convenience while factor 2 addressed TC usability. Regarding the IBD-TCSQ-provider questionnaire, the standardized level of TC satisfaction was 5.76 (SD=0.68) on a scale from 1.00 to 7.00.

**Conclusion(s):**

We generated questionnaires to measure satisfaction with TC among individuals living with IBD and GCPs. The study results confirmed good validity and reliability of the IBD-TCSQ-patient questionnaire. The IBD-TCSQ-provider questionnaire was adapted; subsequent studies could assess its validity and reliability among GCPs nationally. These questionnaires could help identify opportunities for improvement and utilization of TC among IBD patients and GCPs.

**Please acknowledge all funding agencies by checking the applicable boxes below:**

Other, None

**Please indicate your source of funding;:**

Saskatchewan Health Research Foundation (SHRF)

**Disclosure of Interest:**

None Declared

